# Application of a universal parasite diagnostic test to biological specimens collected from animals

**DOI:** 10.1016/j.ijppaw.2022.12.003

**Published:** 2022-12-22

**Authors:** Meredith Lane, Mitra Kashani, Joel LN. Barratt, Yvonne Qvarnstrom, Michael J. Yabsley, Kayla B. Garrett, Richard S. Bradbury

**Affiliations:** a Parasitic Diseases Branch, Division of Parasitic Diseases and Malaria, Center for Global Health, Centers for Disease Control and Prevention, Atlanta, GA, USA; b Synergy America Inc., Duluth, GA, USA; c Oak Ridge Institute for Science and Education, Oak Ridge, TN, USA; d Southeastern Cooperative Wildlife Disease Study, Department of Population Health, College of Veterinary Medicine, University of Georgia, Athens, GA, USA; e Warnell School of Forestry and Natural Resources, University of Georgia, Athens, GA, USA; f Center for the Ecology of Infectious Diseases, University of Georgia, Athens, GA, USA; g School of Health and Life Sciences, Federation University, Berwick Campus, Berwick, Victoria, Australia

**Keywords:** Parasite, Diagnosis, High throughput sequencing, Wildlife, Illumina, Veterinary

## Abstract

A previously described universal parasite diagnostic (nUPDx) based on PCR amplification of the 18S rDNA and deep-amplicon sequencing, can detect human blood parasites with a sensitivity comparable to real-time PCR. To date, the efficacy of this assay has only been assessed on human blood. This study assessed the utility of nUPDx for the detection of parasitic infections in animals using blood, tissues, and other biological sample types from mammals, birds, and reptiles, known to be infected with helminth, apicomplexan, or pentastomid parasites (confirmed by microscopy or PCR), as well as negative samples. nUPDx confirmed apicomplexan and/or nematode infections in 24 of 32 parasite-positive mammals, while also identifying several undetected coinfections. nUPDx detected infections in 6 of 13 positive bird and 1 of 2 positive reptile samples. When applied to 10 whole parasite specimens (worms and arthropods), nUPDx identified all to the genus or family level, and detected one incorrect identification made by morphology. *Babesia* sp. infections were detected in 5 of the 13 samples that were negative by other diagnostic approaches. While nUPDx did not detect PCR/microscopy-confirmed trichomonads or amoebae in cloacal swabs/tissue from 8 birds and 2 reptiles due to primer template mismatches, 4 previously undetected apicomplexans were detected in these samples. Future efforts to improve the utility of the assay should focus on validation against a larger panel of tissue types and animal species. Overall, nUPDx shows promise for use in both veterinary diagnostics and wildlife surveillance, especially because species-specific PCRs can miss unknown or unexpected pathogens.

## Highlights

• A universal parasite diagnostic based on Illumina sequencing has been described.
• This test (nUPDx) has only previously been applied to human blood specimens.
• We applied nUPDx to tissues from various animals infected with a range of parasites.
• NUPDx detected several infections not previously detected using microscopy or PCR.

## Graphical abstract

**Figure:**
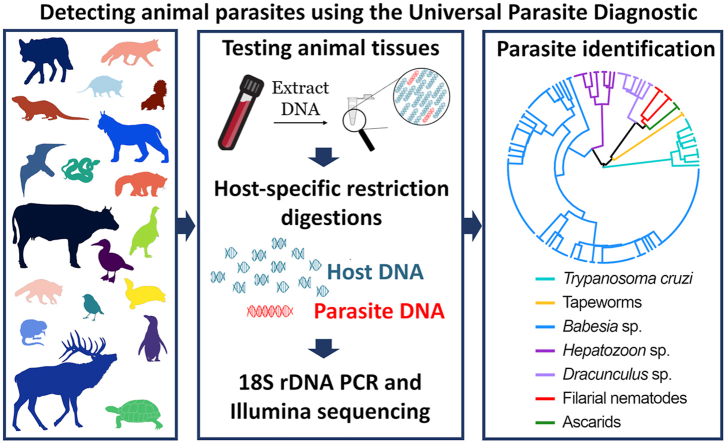

## 1 Background

Diagnosing suspected parasite infections often requires multiple tests, specialized skills (i.e., microscopy/morphology), may lack sensitivity, and requires prescient knowledge of a likely infectious agent to select an appropriate pathogen-specific test (Flaherty et al., 2018, 2021). Microscopy remains the gold standard parasitological diagnostic method, though is considered insensitive compared to other test systems and does not always provide species level differentiation (Lee et al., 2009; Warren et al., 2015). Molecular methods offer a sensitive and specific alternative to microscopy, though arriving at a correct diagnosis may still be challenging; symptoms of parasitic infections are often non-specific and clinicians may not have a sufficiently strong suspicion of the etiology to inform selection of the most appropriate test. Increasingly, metagenomic sequencing is being described as a diagnostic alternative that potentially addresses these bottlenecks (Simner et al., 2018).

The inability to detect parasite coinfections is a limitation of conventional pathogen-specific diagnostic modalities such as PCR, qPCR, and ELISA. Multiplex assays based on the aforementioned test systems can address this to some extent, though multiplex panels are still limited to certain taxa, meaning that rare infections could still be overlooked. Next generation sequencing (NGS) of amplicons derived from highly conserved molecular targets (e.g., eukaryotic 18S rDNA) can facilitate detection of parasite coinfections in a single sample without the need for multiple pathogen-specific tests. NGS may also capture rare and/or unanticipated etiologies. In the context of veterinary medicine, NGS also offers a unique opportunity to uncover previously unappreciated parasitic infections, particularly those of wildlife and exotic pets where a knowledge of parasitic pathogens may be scarce or incomplete. While the costs of NGS methods can be prohibitive (Flaherty et al., 2021), and the lack of reference sequences (i.e., 18S rDNA and others) for obscure parasite taxa (i.e., rare, novel, and emerging zoonoses) may prevent assignment of a precise taxonomic identity to a detected sequence, the methods still show promise for use in diagnostic settings.

Approximately 75% of emerging infectious diseases are caused by zoonoses with 2.5 billion global cases of human illness and 2.7 million global human deaths attributed to zoonoses annually, many of which are derived from wildlife (Salyer et al., 2017). Although the extent to which human morbidity is caused by pet-related zoonoses is unknown, living in close quarters with animals is a recognized risk factor for zoonotic disease (Stull et al., 2015). The National Pet Owners Survey estimates approximately 70% of US households have a pet (Anonymous, 2022), and the causal relationship resulting in pathogen transmission due to living in close contact with animals should be further examined. Parasitic infections of livestock intended for human or pet consumption may also pose a risk to consumers (Davies et al., 2019; Almeria and Dubey, 2021; Rosenthal, 2021). With the decreasing cost of high throughput sequencing (HTS), and the continued risk of rare and unusual zoonotic infections, HTS-based parasite detection may soon become commonplace in clinical settings, in both human medicine and veterinary contexts.

An HTS-based universal parasite diagnostic assay (nUPDx) was recently described that can detect and identify human blood parasites such as *Plasmodium* spp., *Babesia* spp*., Trypanosoma* spp., and several filariases, with a limit of detection comparable to real-time PCR (Flaherty et al., 2021). nUPDx involves nested amplification of the conserved 18S rDNA, followed by Illumina deep-sequencing of the resultant amplicons. This assay can detect various parasites in human blood, including parasite coinfections (Flaherty et al., 2018, 2021; Clemons et al., 2021). Uniquely, nUPDx includes restriction enzyme digestion steps targeting nucleotide polymorphisms that exist within vertebrate 18S rDNA genes that do not exist within 18S rDNA genes of helminths and apicomplexan parasites. This digestion step reduces the proportion of host-derived sequencing reads obtained from a sample, improving parasite detection (Flaherty et al., 2018, 2021). To date, nUPDx and its earlier variants have only been evaluated on selected parasites found in human blood (Flaherty et al., 2018, 2021; Clemons et al., 2021).

The present study sought to investigate the utility of nUPDx when applied to a range of tissues from various animal species, including pets and wildlife, which may be a source of emerging parasitic zoonoses. Blood and tissues from mammals, birds, and reptiles, with a known parasite infection were tested, in addition to specimens that previously tested negative using pathogen-specific tests. While nUPDx lacked sensitivity compared to certain pathogen-specific molecular tests, it detected several unanticipated coinfections, in addition to infections in animals that previously tested negative using other assays. These results suggest that future efforts to improve nUPDx should focus on increasing its sensitivity in animal specimens. Regardless, nUPDx showed great promise as a screening tool for detecting apicomplexans and helminths in various tissues from animals, particularly mammals.

## 2 Methods

### 2.1 Specimens

This study utilized 85 DNA extracts from various tissues obtained from numerous animals (mammals, birds, reptiles) and some gross/whole parasite specimens derived from 80 animals in total. These DNA extracts were prepared as part of the Southeastern Cooperative Wildlife Disease Study (SCWDS) (University of Georgia, College of Veterinary Medicine, Athens, Georgia, USA). Detailed descriptions of these specimens are provided in Table 1, Table 2, Table 3, Table 4, Table 5. DNA was extracted using a Qiagen Dneasy Blood and Tissue extraction kit (Qiagen, Germantown, Maryland, USA) following the manufacturer's instructions. The diagnostic methods used to confirm parasite infections are shown in Table 1, Table 2, Table 3, Table 4, Table 5. Four whole blood samples from parasite-free human donors were used as negative controls for PCR and sequencing. DNA was extracted from the four 200 μL aliquots of parasite-free whole human blood using a Qiagen QIAcube and a QIAmp DNA Blood Mini QIAcube kit (Qiagen) with a custom low-elution volume protocol resulting in DNA eluates of 50 μL, as previously described (Flaherty et al., 2021).

**Table 1:** Parasite-positive^a^ tissues collected from mammals (n = 32).

Specimen Name	Host	Parasite detected previously^a^	Tissue	Previous detection method^a^	nUPDx result^c^	Reference
Cow 1	Domestic cow (*Bos taurus*)	*Theileria orientalis* IKEDA strain	Blood	PCR/sequencing/microscopy	*Theileria* sp.	Oakes et al. (2019)
Dog 1	Domestic dog (*Canis lupus familiaris*)	*Porocephalus crotali*	Lymph node	PCR and morphology	Negative^d^	SCWDS§
Elk 2 (liv)	Elk (*Cervus canadensis*)	*Theileria* sp. (related to *T. cervi*) of white-tailed deer	Liver	PCR/sequencing	*Theileria* spp.	SCWDS diagnostic case using methods in Thompson et al. (2022)
Elk 2 (sp)	Elk	*Theileria* sp. (related to *T. cervi*) of white-tailed deer	Spleen	PCR/sequencing	*Theileria* spp.	SCWDS diagnostic case using methods in Thompson et al. (2022)
Gray Fox 2	Gray fox (*Urocyon cinereoargenteus*)	*Babesia vulpes*	Spleen	PCR	Negative	SCWDS§
Lynx 1	Canada lynx (*Lynx canadensis*)	*Hepatozoon* sp.	Blood	PCR	Negative	SCWDS§
Lynx 2	Canada lynx	*Hepatozoon* sp.	Blood	PCR	Negative	SCWDS§
Lynx 3	Canada lynx	*Hepatozoon* sp.	Blood	PCR	*Hepatozoon felis*	SCWDS§
Mule Deer 1	Mule deer (*Odocoileus hemionus*)	*Babesia* sp.	Spleen	PCR	Negative	Thompson et al. (2022)
Muskrat 1	Muskrat (*Ondatra zibethicus*)	*Hydatigera taeniaeformis*	Liver nodules	PCR and morphology	*Hydatigera* sp.	Ganoe et al. (2021)
Raccoon 1	Northern raccoon (*Procyon lotor*)	*Babesia microti-like*	Spleen	PCR	*Babesia* spp.	Garrett et al. (2019)
Raccoon 11	Northern raccoon	*Babesia* (co-inf.)	Spleen	PCR	*Babesia* spp. +	Garrett et al. (2019)
Raccoon 17	Northern raccoon	*Babesia sensu stricto*	Blood	PCR	*Babesia* spp. +	Garrett et al. (2019)
Raccoon 18	Northern raccoon	*Babesia microti*-like	Blood	PCR	Negative	Garrett et al. (2019)
Raccoon 19	Northern raccoon	*Babesia* (co-inf.)	Spleen	PCR	*Babesia* spp. & a filarial nematode	Garrett et al. (2019)
Raccoon 2	Northern raccoon	*Babesia* (co-inf.)	Spleen	PCR	*Babesia* spp. +	Garrett et al. (2019)
Raccoon 20	Northern raccoon	*Babesia microti*-like	Spleen	PCR	*Babesia* spp. & *Hepatozoon* sp.	Garrett et al. (2019)
Raccoon 21	Northern raccoon	*Babesia sensu stricto*	Blood	PCR	*Babesia* spp. +	Garrett et al. (2019)
Raccoon 22	Northern raccoon	*Babesia microti*-like	Blood	PCR	*Babesia* spp.	Garrett et al. (2019)
Raccoon 23	Northern raccoon	*Babesia microti*-like	Spleen	PCR	Negative	Garrett et al. (2019)
Raccoon 25 (replicate 1)^b^	Northern raccoon	*Babesia* (co-inf.)	Spleen	PCR	*Babesia* spp.	Garrett et al. (2019)
Raccoon 25 (replicate 2)^b^	Northern raccoon	*Babesia* (co-inf.)	Spleen	PCR	*Babesia* spp.	Garrett et al. (2019)
Raccoon 3	Northern raccoon	*Babesia sensu stricto*	Blood	PCR	*Babesia* sp.	Garrett et al. (2019)
Raccoon 4	Northern raccoon	*Babesia microti*-like	Blood	PCR	*Babesia* sp.	Garrett et al. (2019)
					*Hepatozoon* sp.	
					Filarial nematode	
Raccoon 5	Northern raccoon	*Babesia* (co-inf.)	Blood	PCR	*Babesia* spp.	Garrett et al. (2019)
Raccoon 7	Northern raccoon	*Babesia sensu stricto*	Blood	PCR	*Babesia* spp. e	Garrett et al. (2019)
Raccoon 8	Northern raccoon	*Babesia microti*-like	Blood	PCR	*Babesia* spp.	Garrett et al. (2019)
Raccoon 9	Northern raccoon	*Babesia microti*-like	Spleen	PCR	*Babesia* sp.	Garrett et al. (2019)
Red Fox 1	Red fox (*Vulpes vulpes*)	*Babesia microti*-like	Spleen	PCR	*Babesia* sp.	SCWDS^f^
					*Hepatozoon* sp.	
Red Panda 1	Red panda (*Ailurus fulgens*)	*Trypanosoma cruzi*	Blood	PCR and microscopy	*Trypanosoma cruzi*	Huckins et al. (2019)
River Otter 1	North American river otter (*Lontra canadensis*)	*Babesia microti*-like	Spleen	PCR	*Babesia* sp.	Garrett et al. (2022)
Striped Skunk 1	Striped skunk (*Mephitis mephitis*)	*Babesia microti*-like	Spleen	PCR	*Babesia* sp.	SCWDS diagnostic case using methods in Garrett et al. (2022)
Striped Skunk 4	Striped skunk	*Babesia microti*-like	Spleen	PCR	*Babesia* sp.	SCWDS diagnostic case using methods in Garrett et al. (2022)
White Tailed Deer 1	White-tailed deer (*Odocoileus virginianus*)	*Sarcocystis* sp.	Muscle	Histology	Negative	SCWDS^f^

Co-inf. = co-infections with multiple species.

a Parasites detected previously using a method listed in this table (parasite-specific PCR or microscopy).

b Two replicates of the same spleen sample were sequenced.

c Complete BLASTN results and the sequences detected are provided in Supplementary file S3.

d *Porocephalus crotali* is a parasitic pentastomid crustacean.

e In these specimens, we detected sequences that were a match to several sequences assigned to *Babesia* in addition to a sequence in GenBank with the accession number MN296295.1. This sequence is listed in GenBank as belonging to *Ixodes ricinus,* which we believe is incorrect. BLASTN searches and alignments clearly show that sequence MN296295.1 belongs to *Babesia* sp., and not to *Ixodes ricinus*. Across 712 overlapping bases, this sequence is identical to sequences submitted to GenBank for *Babesia capreoli* (e.g., GenBank Accession: KX839234.1) and is >99% identical to sequence in GenBank assigned to other *Babesia* species.

f Specimen is from a Southeastern Cooperative Wildlife Disease Study (SCWDS) veterinary case and no published reference studies are available.

**Table 2:** Bird (n = 13) and reptile (n = 2) specimens positive for apicomplexan parasites^a^

Specimen name	Host	Parasite detected previously^a^	Previous detection method (reference)^a^	Tissue	nUPDx result^c^	References
Bluebird 1	Eastern bluebird (*Sialia sialis*)	*Sarcocystis* sp. (likely *S. speeri*)	PCR/sequencing/histology	Skeletal muscle	Negative	Carleton et al. (2012)
Duck 1	Khaki Campbell duck *(Anas platyrhynchos domesticus*)	*Leucocytozoon* sp.	PCR/sequencing	Blood	Negative	SCWDS^b^
Owl 1	Great horned owl (*Bubo virginianus*)	*Leucocytozoon* sp.	PCR/sequencing/microscopy	Liver	Negative	Niedringhaus et al. (2018)
Owl 2	Great horned owl	*Leucocytozoon* sp.	PCR/sequencing/microscopy	Liver	Negative	Niedringhaus et al. (2018)
Penguin 1	Little penguin (*Eudyptula minor*)	*Plasmodium* sp. lineage LINN1	PCR/sequencing	Spleen	Negative	SCWDS^b^
Gannet 2 (H)	Northern gannet (*Morus bassanus*)	*Sarcocystis falcatula*	PCR/sequencing/histology	Heart (H)	*Sarcocystis* sp.	SCWDS^b^
Gannet 2 (M)	Northern gannet	*Sarcocystis falcatula*	PCR/sequencing/histology	Skeletal muscle (M)	*Sarcocystis* sp.	SCWDS^b^
Gannet 3 (H)	Northern gannet	*Sarcocystis falcatula*	PCR/sequencing/histology	Heart	*Sarcocystis* sp.	SCWDS^b^
Gannet 3 (M)	Northern gannet	*Sarcocystis falcatula*	PCR/sequencing/histology	Skeletal muscle	*Sarcocystis* sp.	SCWDS^b^
Gannet 4 (H)	Northern gannet	*Sarcocystis falcatula*	PCR/sequencing/histology	Heart	*Sarcocystis* sp.	SCWDS^b^
Gannet 4 (M)	Northern gannet	*Sarcocystis falcatula*	PCR/sequencing/histology	Skeletal muscle	*Sarcocystis* sp.	SCWDS^b^
Pygmy Falcon 1	Pygmy falcon (*Polihierax semitorquatus)*	*Plasmodium* sp. pZEMAC01	PCR/sequencing	Spleen	*Plasmodium* sp.	SCWDS^b^
Pygmy Falcon 2	Pygmy falcon	*Plasmodium* sp. pZEMAC01	PCR/sequencing	Liver	*Plasmodium* sp.	SCWDS^b^
Turkey 7	Wild turkey (*Meleagris gallopavo)*	*Toxoplasma gondii*	PCR/sequencing/histology (immunohistochemistry)	Lung	*Toxoplasma gondii* or other Toxoplasmatinae	SCWDS^b^
Turkey Vulture 1	Turkey vulture (*Cathartes aura*)	*Haemoproteus catharti*	PCR/sequencing/microscopy	Blood	Negative	Yabsley et al. (2018)
Turkey Vulture 2	Turkey vulture	*Haemoproteus catharti*	PCR/sequencing	Blood	Negative	Yabsley et al. (2018)
Tortoise 1	Gopher tortoise (*Gopherus polyphemus)*	*Hepatozoon* sp.	PCR/sequencing/microscopy	Blood	*Hepatozoon* sp.	McGuire et al. (2014)
Tortoise 2	Gopher tortoise	*Hepatozoon* sp.	PCR/sequencing/microscopy	Blood	Negative	McGuire et al. (2014)

a Parasites detected previously using a method listed in this table (usually a parasite-specific PCR or microscopy). A reference is provided if available.

b Specimen detected in Southeastern Cooperative Wildlife Disease Study (SCWDS) veterinary or diagnostic case and no published reference studies are available.

c Complete BLASTN results and the sequences detected are provided in Supplementary file S1.

**Table 3:** Parasite-negative specimens from 12 mammals and a single bird^a^.

Specimen name	Host	Parasite detected previously	Tissue	Test employed to indicate negative status	nUPDx result^d^	References
Dog 2	Domestic dog *(Canis lupus familiaris)*	negative	Blood	Microscopy	Negative	Confirmed by CDC parasitology reference lab.
Dog 3	Domestic dog	negative	Blood	Microscopy	Negative	Confirmed by CDC parasitology reference lab.
Dog 4	Domestic dog	negative	Blood	Microscopy	Negative	Confirmed by CDC parasitology reference lab.
Opossum 1	Virginia opossum (*Didelphis virginiana*)	negative	Blood	PCR for *Babesia* spp.	Negative	SCWDS^b^
Raccoon 13	Northern raccoon (*Procyon lotor)*	negative	Spleen	PCR for *Babesia* spp.	*Babesia* sp.	Garrett et al. (2019)
Raccoon 14	Northern raccoon	negative	Spleen	PCR for *Babesia* spp.	*Babesia* sp.^c^	Garrett et al. (2019)
Raccoon 27	Northern raccoon	negative	Blood	PCR for *Babesia* spp.	*Babesia* sp.	Garrett et al. (2019)
Raccoon 28	Northern raccoon	negative	Spleen	PCR for *Babesia* spp.	*Babesia* sp.	Garrett et al. (2019)
Raccoon 29	Northern raccoon	negative	Spleen	PCR for *Babesia* spp.	*Babesia* sp.	Garrett et al. (2019)
Raccoon 33	Northern raccoon	negative	Blood	PCR for *Babesia* spp.	Negative	Garrett et al. (2019)
Striped Skunk 2	Striped skunk (*Mephitis mephitis)*	negative	Blood	PCR for *Babesia* spp.	Negative	Garrett et al. (2022)
Striped Skunk 3	Striped skunk	negative	Blood	PCR for *Babesia* spp.	Negative	Garrett et al. (2022)
Black Vulture 1	Black vulture *(Coragyps atratus)*	negative	Blood	PCR	Negative	Yabsley et al. (2018)

a For these samples, the negative result was based on a screen using a single PCR assay for one target organism only or by microscopic examination of the specimen, so this does not exclude that these specimens may have been positive for other off-target parasitic pathogens. Table excludes the four blood samples from a human donor that were included in this study as true negative controls.

b Specimen detected in Southeastern Cooperative Wildlife Disease Study (SCWDS) veterinary case and no published reference studies are available.

c Equivocal. Right on the cutoff threshold of 20 reads.

d Complete BLASTN results and the sequences detected are provided in Supplementary file S1.

**Table 4:** Specimens from birds (n = 8) and reptiles (n = 2) positive for amoebae or trichomonads.

Specimen name	Host	Parasite detected previously^b^	Matrix or tissue	Previous detection method (reference)^b^	nUPDx result^c^
Eagle 1	Bald eagle (*Haliaeetus leucocephalus)*	*Trichomonas* sp.	Cloacal swab	PCR/sequencing	Negative
Eagle 2	Bald eagle	*Trichomonas* sp.	Cloacal swab	PCR/sequencing	Negative
Turkey 1	Wild turkey (*Meleagris gallopavo*)	*Tetratrichomonas* sp.	Caecum	PCR/sequencing	Undescribed apicomplexan^a^
Turkey 2	Wild turkey	*Tetratrichomonas* sp.	Caecum	PCR/sequencing	Undescribed alveolate and/or apicomplexan^a^
Turkey 3	Wild turkey	*Tetratrichomonas* sp.	Caecum	PCR/sequencing	Negative
Turkey 4	Wild turkey	*Tetratrichomonas* sp.	Caecum	PCR/sequencing	Undescribed apicomplexan^a^
Turkey 5	Wild turkey	*Histomonas meleagris*	Liver	PCR/sequencing	Negative
Turkey 6	Wild turkey	*Histomonas meleagris*	Liver	PCR/sequencing	Negative
Turtle 1	Florida softshell turtle *(Apalone ferox*)	*Entamoeba* sp.	Spleen	Morphology/microscopy	Negative
Turtle 2	Florida softshell turtle	*Entamoeba* sp.	Spleen	Morphology/microscopy	*Haemogregarina* sp.

Note: Specimen detected in Southeastern Cooperative Wildlife Disease Study (SCWDS) veterinary case and no published reference studies are available.

*Two replicates of the same blood sample were sequenced.

a BLASTN hits to *Monocystis agilis* and/or uncultured alveolate were obtained in these samples. *Monocystis* is a parasite of earthworms and detection of these sequences likely reflect consumption of earthworms by turkeys.

b Parasites detected previously using a method listed in this table.

c Complete BLASTN results and the sequences detected are provided in Supplementary file S1.

**Table 5:** Whole parasites from mammals, birds and reptiles (n = 10) tested using nUPDx.

Specimen name	Host	Parasite	Specimen description	nUPDx result^b^	Reference
Coyote 1	Coyote (*Canis latrans*)	*Dirofilaria immitis*	Whole worm	*Dirofilaria immitis*	SCWDS^e^
Coyote 2	Coyote	*Dirofilaria immitis*	Whole worm	*Dirofilaria immitis*	SCWDS^e^
Opossum 2	Virginia opossum (*Didelphis virginiana*)	*Dracunculus insignis*	Whole worm	*Dracunculus* sp.	Cleveland et al. (2020)
Raccoon 31	Northern raccoon (*Procyon lotor)*	*Baylisascaris procyonis*	Whole worm	Nematode (Ascarididae sp.)	Sapp et al. (2020)
Raccoon 32	Raccoon	*Baylisascaris procyonis*	Whole worm	Nematode (Ascarididae sp.)	Sapp et al. (2020)
Raccoon 34	Raccoon	*Dracunculus insignis*	Whole worm	*Dracunculus* sp.	Cleveland et al. (2020)
Robin 1	European robin (*Erithacus rubecula)*	*Aprocta cylindrica*	Whole worm	Nematode (Sprurina sp.)	SCWDS^e^
Penguin 10	Magellanic penguin (*Spheniscus magellanicus*)	nasal mite^a^	Whole mite	Mite (Mesostigmata sp.)	Specimens collected in study by Vanstreels et al. (2019)
Penguin 9	Magellanic penguin	nasal mite^a^	Whole mite	Tick (Ixodida sp.)^d^	Specimens collected in study by Vanstreels et al. (2019)
Snake 1	Red-bellied watersnake (*Nerodia erythrogaster*)	*Ophidascaris* sp.	Whole worm	Nematode (Ascarididae sp.) and *Hepatozoon* sp.^c^	SCWDS^e^

a Species of nasal mite is unknown.

b Complete BLASTN results and the sequences detected are provided in Supplementary file S1, Tab.

c It was assumed that the snake host was infected with a *Hepatozoon* sp. and the DNA from this organism was also detected within the DNA extract of the whole worm.

d For this specimen, UPDx led to reassignment of the organism's originally assigned identity.

e Specimen detected in Southeastern Cooperative Wildlife Disease Study (SCWDS) veterinary case and no published reference studies are available.

### 2.2 nUPDx for detection of parasites in animal tissues

**Fig. 1:**
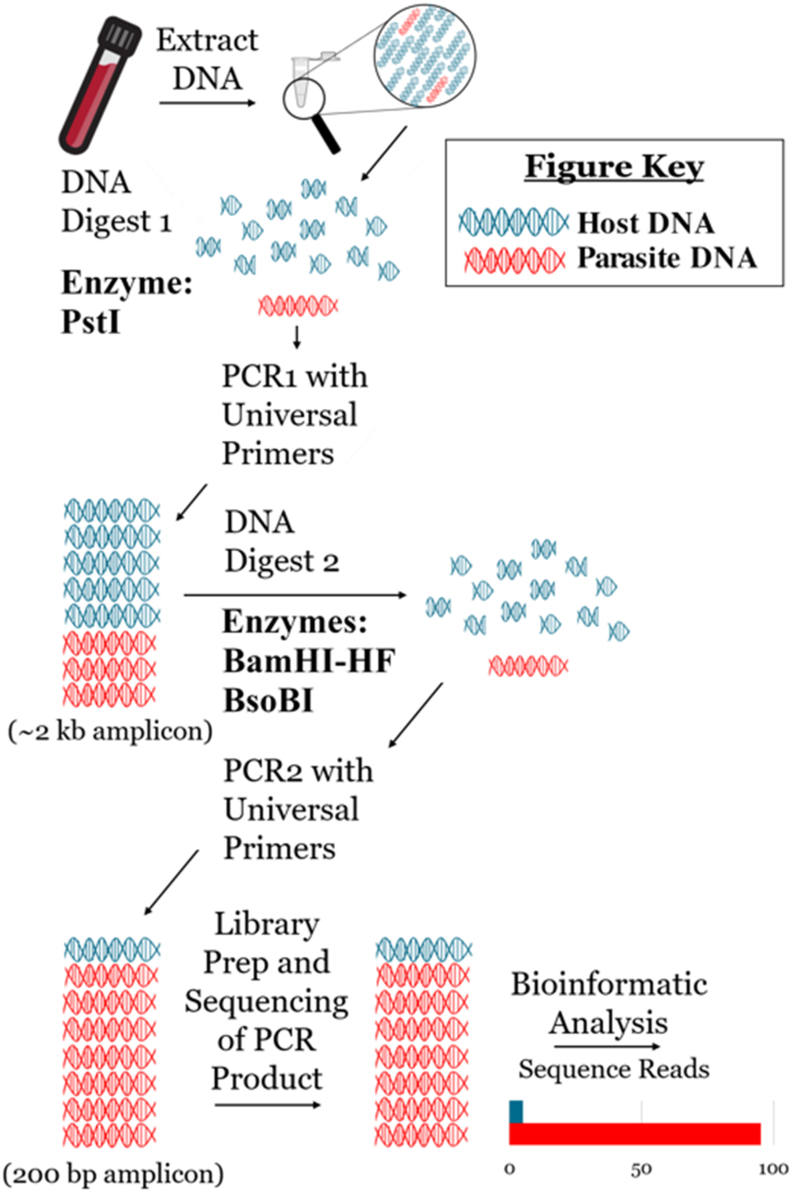
Schematic describing the nested UPDx protocol employed in this study. This schematic provides a summary of the nested UPDx (nUPDx) protocol originally described by Flaherty et al. (Flaherty et al., 2021). Briefly, the DNA extract is subjected to a restriction digestion using the *Pst*I restriction enzyme, and the digest product is subjected to PCR1 using primers 5’TTGATCCTGCCAGTAGTCATATGC’3 (outer forward) and 5’GGTGTGTACAAAGGGCAGGGAC’3 (outer reverse). The resultant ∼2 kb amplicon is digested using the restriction enzymes *Bam*HI and *Bso*BI. The digest product is then subjected to PCR2 using internal primers 5’CCGGAGAGGGAGCCTGAGA’3 (inner forward) and 5’GAGCTGGAATTACCGCGG’3 (inner reverse) originally described by Flaherty et al. (Flaherty et al., 2018, 2021). The amplicon of PCR2 (∼200 base pairs) is finally subjected to Illumina amplicon sequencing.

Amplicons were generated from the DNA extracts using the nested UPDx (nUPDx) assay described by Flaherty et al. (2021) and in Fig. 1. Briefly, extracted DNA is digested using the *Pst*I restriction enzyme, followed by PCR amplification of part of the 18S rDNA locus using the PCR1 forward primer 5’TTGATCCTGCCAGTAGTCATATGC’3 and the PCR1 reverse primer 5’GGTGTGTACAAAGGGCAGGGAC’3. The resultant ∼2 kb amplicon is digested using the *Bam*HI and *Bso*BI restriction enzymes. The resulting digested product is then subjected to a second PCR using the PCR2 forward primer 5’CCGGAGAGGGAGCCTGAGA’3 and the PCR2 reverse primer 5’GAGCTGGAATTACCGCGG’3. Amplicons were analyzed via agarose gel electrophoresis (1.5% gel) to confirm amplification prior to sequencing. These amplicons were purified using a Monarch PCR & DNA Cleanup Kit (<2 kb) (New England Biolabs, Ipswich, MA, USA), and the DNA concentration of purified eluates was determined using a Qubit® 2.0 Fluorometer and Qubit® dsDNA High Sensitivity (HS) Assay Kit (Waltham, MA, USA). Illumina libraries were constructed from the purified amplicons using the NEBNext Ultra Library Prep Kit for Illumina and the NEBNext Multiplex Oligos for Illumina (96 index primers) (New England Biolabs). Note that steps without size selection were executed during library preparation, in accordance with the manufacturer's instructions (New England Biolabs). All library preparation wash steps were conducted using AMPure XP Beads (Beckman Coulter, Brea, CA, USA). Following library preparation, the DNA concentration of individual barcoded samples was determined using the Qubit as described above. Based on the Qubit results, all samples were subsequently normalized by diluting samples to the same concentration as the sample with the lowest concentration. Normalized barcoded samples were then pooled by adding the same volume of each barcoded sample to a single tube. The DNA concentration of the pooled library was measured using the Qubit once more, to check for consistency, and the average DNA fragment length was determined using the Agilent Tapestation 2200 with D1000 ScreenTape system (Agilent, Santa Clara, California, USA). Next, the library pool was denatured using NaOH and diluted to a final loading concentration of 10 pM, in accordance with the Illumina Miseq User Guide and sequenced on the Illumina Miseq platform using Miseq Reagent V2 Nano Kits (500 cycles) (Illumina). A 10% spike of PhiX Control v3 (Illumina) was included with each sequencing run. Illumina sequencing reads for each specimen are available in NCBI under BioProject accession number PRJNA437674. BioSamples and SRA reads within this BioProject that were uploaded as part of the present study are labelled with the same specimen names as provided in Table 1, Table 2, Table 3, Table 4, Table 5 and in Supplementary File S3.

### 2.3 Bioinformatic analysis of illumina data

Following sequencing, the resultant fastq files generated for each animal sample (n = 86) and the human-derived negative control blood samples (n = 4), were imported into the Geneious Prime console (Biomatters, Ltd. NZ) and subjected to the custom workflow originally described by Flaherty et al. (2021), though with modifications to the reference sequence database to include a variety of vertebrate 18S rDNA sequences from non-human mammals, birds, and reptiles and to include a broader range of parasite-derived 18S rDNA sequences obtained from GenBank. Sequences are provided in Supplementary Files S1 and S2.

Briefly, our custom Geneious workflow (Geneious Prime: www.geneious.com) first removed the nUPDx primer sequences from either end of the Illumina reads (250 bases, paired end) using the Trim Ends plugin, allowing 3 mismatches and a minimum match length of 5 bases. BBDuk was then used to remove Illumina adapter sequences. Low-quality bases were trimmed from either end of the reads (minimum Phred score of 20) and remaining reads shorter than 50 bases in length were discarded. Paired reads were then merged using BBmerge (within the Geneious interface) with default parameters. Remaining reads were subjected to a BLASTN search against vertebrate 18S rDNA sequences (Supplementary File S1) using a percent identity of 99%, a word size of 11, and a qcov_hsp_perc value of 60. Reads obtaining a match to the host exclusion database using these parameters were discarded. Remaining reads were assembled using the Geneious de novo assembler using a minimum overlap of 50 bases and a minimum overlap identity of 100% (all other values set to default). The resulting haplotypes were next subjected to a BLASTN search (default parameters) against our updated database of parasite-derived 18S sequences (Supplementary Files S2), and the nearest BLASTN match result for each sequence was exported to text. To establish a threshold for distinguishing between positive and negative results, we employed the system described by Flaherty et al. (2021), where the number of merged reads used to construct a detected parasite-derived haplotype was calculated as a proportion of the total number of merged reads generated for that specimen. The proportion of parasite-derived reads was then used to determine the specimens’ status as positive or negative for a given parasite, based on a threshold value computed using the four parasite-negative human blood samples, as previously described (Flaherty et al., 2018, 2021).

### 2.4 Sequence clustering and tree rendering

The parasite-derived sequences detected were first aligned using MUSCLE in the Geneious Prime console and exported from Geneious as an alignment (a text file) in FASTA file format. Dendrograms were constructed from the resultant alignments in R (version 4.1.2, R Core Team, 2021). The fasta alignment was imported into R as an “msa” object using the “msa” package (Bodenhofer et al., 2015), and the aligned sequences were used to compute a pairwise distance matrix using the “seqinr” R package (Version 4.2.8) (Charif and Lobry, 2007). The matrix was clustered using hierarchical agglomerative nesting (AGNES) via the “cluster” package (Version 2.1.2; Maechler et al., 2022), which employed the “average” clustering method with all other parameters set to default. The resulting dendrograms were visualized using the ‘ggtree’ package (Version 3.2.1) (Yu, 2020). Animal silhouettes used to annotate trees were obtained from phylopic.org or were made by the authors using the GIMP image manipulation suite.

### 2.5 Statistical analysis

We used Fisher's exact tests to determine if the status of a nUPDx test result (positive or negative) depended on whether the host animal was a bird/reptile or a mammal. This analysis considered birds and reptiles as a single group, as it was postulated that a reduction in nUPDx sensitivity might be observed when the assay is applied to birds and reptiles because they possess nucleated red blood cells (RBCs) while mammals possess anucleate RBCs. This might plausibly increase the proportion of host-derived DNA in tissue/blood DNA extracts collected from birds and reptiles relative to mammals, potentially reducing nUPDx sensitivity when testing the former group. We also used Fisher's exact test to determine whether a nUPDx test result (positive or negative) was dependent to some extent on whether the sample being tested was a spleen sample or a blood sample. Too few specimens were available from other anatomical sites for additional analysis.

## 3 Results

### 3.1 Computation of the coverage threshold for specimen positivity

In accordance with the methods described by Flaherty et al. for determining the positive or negative status of an nUPDx test (Flaherty et al., 2018, 2021), we sequenced four specimens comprised of parasite-negative human blood. These negatives were sequenced to establish a background frequency of parasite-derived reads that may be observed in fastq files of negative specimens as a consequence of index cross-talk (i.e., bleed through), as opposed to laboratory contamination, or the true presence of parasite DNA in the specimen (Flaherty et al., 2018, 2021). The fastq files generated from these four negatives each contained no parasite derived reads (i.e., no evidence of index cross-talk). Consequently, in accordance with Flaherty et al., 2018, 2021, the threshold for positivity was set to 20, such that more than 20 merged parasite-derived reads were required before a sample was considered positive for a given parasite. Note that if low-level index cross-talk had been detected, a dynamic cutoff threshold would have been calculated based on the specific proportion of parasite-derived reads detected in these negatives, as described by Flaherty et al. (2018). The absence of parasite-derived reads in these negative specimens reflected a lack of laboratory contamination and a lack of index cross-talk, allowing us to proceed to bioinformatic analysis using 20 reads as our threshold for positivity as described by Flaherty et al. (2018).

### 3.2 Parasite-positive tissues collected from mammals

**Fig. 2:**
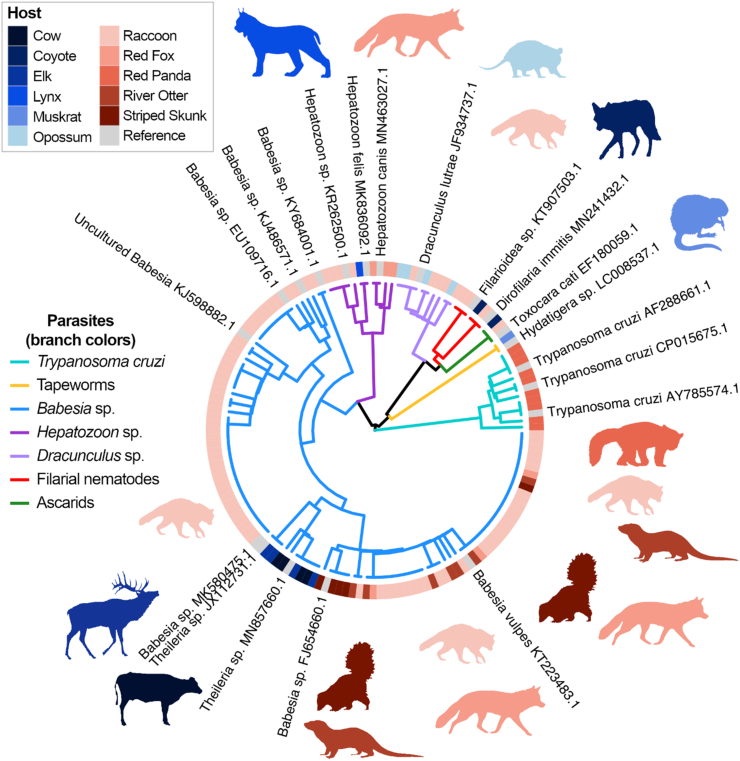
Cluster dendrogram showing parasite species detected in mammalian hosts. Sequences detected in each specimen using nUPDx were clustered alongside parasite-derived reference sequences of known identity obtained from GenBank. These reference sequences are labelled on the dendrogram branch tips (where appropriate). A peripheral color-coded heat map ring indicates the host animal from which the parasite-derived sequence was detected. Gray branches and blocks on the heat map reflect the position of reference sequences within the tree. (For interpretation of the references to color in this figure legend, the reader is referred to the Web version of this article.)

Of the 32 parasite-positive mammals tested, nUPDx detected parasites in 24 of these (75%) (Table 1). However, nUPDx was also able to detect several unanticipated co-infections in these specimens. For instance, Raccoon 4 was known to be positive for a *Babesia microti*-like parasite, yet was found to be positive for *Babesia* sp., *Hepatozoon* sp., and a filarial nematode via nUPDx. Similarly, Raccoon 19 had a known *Babesia* spp. coinfection, but nUPDx also detected a concurrent filarial nematode. Similarly, in Red Fox 1, which was known to be infected with a *Babesia vulpes*-like sp., nUPDx confirmed the *Babesia* sp. infection in addition to a *Hepatozoon* sp. infection (Table 1). Several of these specimens were known to be positive for multiple *Babesia* species (Table 1) and, in all cases, nUPDx supported this multi-species diagnosis. There were also several instances where nUPDx detected *Babesia* spp. (i.e., multiple species), whereas the original diagnosis supported infection with a single species (Table 1). Notably, while nUPDx was able to detect infections with apicomplexan parasites and helminths (i.e., filarial nematodes and a tapeworm: *Hydatigera* sp.), nUPDx failed to detect the infection caused by the parasitic pentastomid *Porocephalus crotali* in a dog. Overall, positive parasite detections were confirmed using nUPDx in a range of tissue matrices including blood (11 of 14; 79%), and solid tissues such as the spleen (12 of 15; 80%) and liver/liver nodules (2 of 2; 100%). A dendrogram displaying the results of a clustering analysis of the sequences detected in these specimens is shown in Fig. 2. Reference sequences obtained from GenBank are also included in the clustering analysis in Fig. 2 to demonstrate the ability of nUPDx to differentiate between certain parasite taxa. Note, however, that identical clustering of a sequence from this study alongside a reference sequence from a known parasite does not necessarily indicate that the animal-derived sequence belongs to the same parasite species as the reference.

### 3.3 Apicomplexan-positive tissues collected from birds and reptiles

**Fig. 3:**
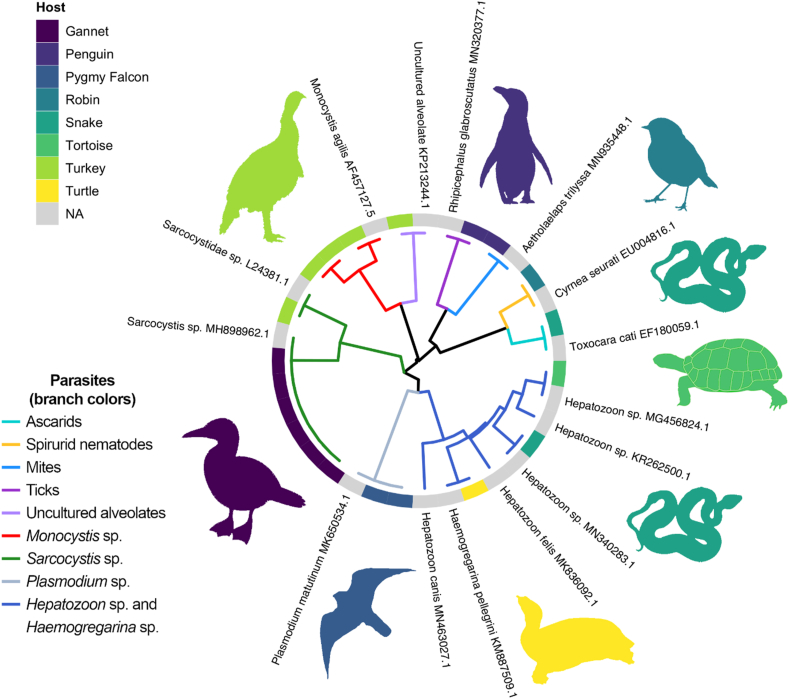
Cluster dendrogram showing parasite species detected in avian and reptilian hosts. Sequences detected in each specimen using nUPDx were clustered in this dendrogram alongside parasite-derived reference sequences of known identity obtained from GenBank. These reference sequences are labelled on the dendrogram branch tips (where appropriate). A peripheral color-coded heat map ring indicates the host animal from which the parasite-derived sequence was detected. Gray branches and blocks on the heat map reflect the position of reference sequences within the tree. (For interpretation of the references to color in this figure legend, the reader is referred to the Web version of this article.)

Of the 13 birds and two reptiles known to be infected with an apicomplexan parasite (Table 2), only 7 of these (1 reptile and 6 birds; ∼54%) tested positive using nUPDx. nUPDx did not detect any coinfections in these samples (Table 2). Positive detections were observed in a range of tissues including blood (1 of 5; 17%), lung tissue (1 of 1; 100%), skeletal muscle (3 of 4; 75%), cardiac muscle (3 of 3; 100%), liver tissue (1 of 3; 33%), and spleen (1 of 2; 50%). A dendrogram displaying the results of a clustering analysis of the sequences detected in these specimens is shown in Fig. 3.

### 3.4 Positive parasite detections in presumably negative mammalian and bird tissue samples

Of the single bird and 12 mammalian samples that previously tested negative for parasites (Table 3), 5 of these (∼38%) tested positive for parasites (*Babesia* sp.) using nUPDx. Positive detections were observed in spleen samples (4 of 4; 100%), and blood samples (1 of 9; 11%). A dendrogram displaying the results of a clustering analysis of the sequences detected in these specimens is shown in Fig. 2.

### 3.5 Application of nUPDx to specimens containing trichomonads and amoebae

Of the trichomonad/amoeba positive specimens from birds (n = 8) and reptiles (n = 2), nUPDx did not detect DNA from either parasite taxa. This was anticipated due to known template-primer mismatches in published 18S rDNA sequences available in the GenBank nucleotide database for all trichomonad and *Entamoeba* species that infect humans and animals. However, nUPDx detected apicomplexan parasites in 4 specimens, though the precise identity of 3 of these apicomplexa (i.e., detected in Turkeys 1, 2 and 4) could not be completely elucidated given available data. However, BLASTN hits obtained for sequences from Turkeys 1, 2 and 4 included matches to *Monocystis agilis,* which is a coccidian parasite of earthworms. Consequently, this may be a case of pseudoparasitism, where detection of these organisms more likely reflects consumption of earthworms by these turkeys (Table 4). The fourth positive apicomplexan detection (i.e., in the spleen of Turtle 1) was of a *Haemogregarina* species (Table 4). A dendrogram displaying the results of a clustering analysis of the sequences detected in these specimens is shown in Fig. 3.

### 3.6 Application of nUPDx to whole/gross parasite specimens

For each of these 10 specimens, nUPDx generated a sequence, that either confirmed the morphologic identification or facilitated identification of the parasite, though usually to the taxonomic level of genus or even order (e.g., ascarids from coyotes and raccoons, and spirurid from robin) (Table 5). In one case, nUPDx led to the identity of the original gross specimen being reassigned; a specimen originally designated as a nasal mite from a magellanic penguin (i.e., Penguin 9) was found to be a tick. Finally, one nematode from a snake was also positive for a protozoan (*Hepatozoon* sp.) that likely was detected in host tissue present on the nematode. Fig. 2, Fig. 3 show a clustering analysis of the sequences obtained from these specimens and demonstrate the ability of nUPDx to differentiate between various parasite taxa.

### 3.7 Association of detection success with specimen type

Fisher's exact tests were performed to explore whether the positive status of a nUPDx result depended on the type of animal being tested. We found that when testing any given tissue type, the positive or negative status of a nUPDx result did not depend significantly (*P* = 0.96) on whether the animal was a mammal (anucleate RBCs) or a bird/reptile (nucleated RBCs), regardless of the tissue being tested (Table 6). If we consider the same comparison (mammal vs. birds/reptiles) and only examine nUPDx results obtained when testing blood, the dependency was also insignificant according to Fisher's exact test (*P* = 0.998). However, the positive/negative status of a UPDx result seemed to show some dependance on whether blood or spleen tissue was tested (*P* = 0.02), regardless of the type of animal (Table 7).

**Table 6:** Contingency tables for Fisher's exact test of nUPDx result status (positive or negative) for bird^a^ and reptile^a^ tissues versus mammalian tissues^b^.

	Mammals^b^	Birds & reptiles^a^	Observed totals
**Considering all tissues: blood and solid tissues**			
Positives	25	10	35
Negatives	8	8	16
Observed totals	33	18	51
Fisher's *P*-value^c^	0.96 – not significant		
**Considering only blood samples**			
Positives	11	1	12
Negatives	3	4	7
Observed totals	14	5	19
Fisher's *P*-value^c^	0.998 – not significant		

Note: This analysis considers all specimens listed in Table 1, Table 2 If two different tissues from the same animal were tested, these were considered as separate for the purposes of this analysis.

a Animals with nucleated red blood cells.

b Animals with anucleate red blood cells.

c One-tailed test.

**Table 7:** Contingency table for Fisher's exact test of nUPDx result status (positive or negative) when testing blood samples (n = 29) versus spleen samples (n = 24)^a^.

	Blood	Spleen	Observed totals
Positives	13	18	31
Negatives	15	5	20
Observed totals	28	23	51
Fisher's *P*-value^b^	0.020 - significant		

a This analysis includes all blood and spleen samples listed in Table 1, Table 2, Table 3, Table 4.

b One-tailed test.

## 4 Discussion

This study shows that nUPDx can detect apicomplexan parasites and helminths in various tissues and other biological samples collected from animals. The ability of nUPDx to broadly detect various apicomplexan species and helminths, in addition to coinfections, would be beneficial for estimating the prevalence of parasitic infections in domestic animals and wildlife, and for monitoring the spread of parasites throughout animal populations or to new populations. The nUPDx assay was particularly useful in detecting co-infections within hosts, even in individuals that had not been previously diagnosed with coinfections. Many samples from raccoons from a piroplasm study (Garrett et al., 2019) were included in this study because these hosts are commonly infected with two distinct lineages of *Babesia* and coinfections were common. However, detection of these lineages is complicated and requires a series of multiple PCR assays that are genus-wide or lineage-specific followed by sequence analysis of all amplicons because the ‘lineage-specific’ assays are not 100% specific. Thus, alternative assays for detection of this piroplasm diversity is needed. The nUPDx was able to accurately detect coinfections and even detected infections in several raccoons that had tested negative with our screening PCR assays. In addition, coinfections with *Hepatozoon* and filarial nematodes that were previously unknown to be present were detected which further highlights the utility of nUPDx to detect unexpected pathogens within clinical samples. The dependence of nUPDx positive or negative test status on whether blood or spleen tissue was tested is also a noteworthy observation, where the testing of spleen tissue seemed more likely to result in a positive parasite detection compared to blood (Fisher's *P*-value = 0.020). However, future studies testing the association between nUPDx positivity and tissue type (e.g., spleen or blood) would benefit from a larger sample size to confirm that this association holds true. In any case, a primary function of the spleen is to detect and remove old, compromised, or infected blood cells and other foreign material. Consequently, parasitized RBCs are concentrated in the spleen where they are removed from circulation and subsequently destroyed. The testing of spleen tissue is often not feasible as a diagnostic recommendation for blood parasites due to the invasive nature of obtaining a spleen biopsy or aspirate, though understanding that testing spleen tissue could increase sensitivity for molecular detection of blood parasites may prove useful to investigators performing population-level surveys of blood parasites in animals where tissues from deceased specimens (e.g., specimens provided by hunters) are available.

While nUPDx likely possesses some utility as both a diagnostic test and as a population-level surveillance tool, the present study highlights some of its limitations. Firstly, nUPDx is not entirely universal; as anticipated, it cannot detect trichomonads or intestinal amoebae as demonstrated by our data. Similarly, nUPDx may be ineffective at detecting some pentastomes; a unique group of parasitic crustaceans. This is based on the negative result obtained here for the specimen containing *Porocephalus crotali* and a subsequent examination of pentastome 18S sequences available in GenBank. Unfortunately, an 18S rDNA sequence for *P. crotali* at the region targeted by nUPDx is unavailable in GenBank. However, examination of other pentastome18S rDNA sequences revealed that while the nUPDx primers should amplify a product from pentastome DNA, some pentastome 18S rDNA sequences (e.g., *Armillifer* sp. – GenBank Accession (GB): LC695012.1, and *Porocephalidae* sp. – GB: LC624910.1) possess a *Bam*HI cut site within the nUPDx amplicon, while other pentastomes (e.g., *Raillietiella* sp. - GB: LC695013.1) do not. Based on these observations, it is possible that the 18S rDNA amplicon of *P. crotali* was digested by the *Bam*HI treatment, rendering it undetectable. Furthermore, the forward primer of PCR1 is not a perfect match for the pentastome template sequences we examined (1 or 2 mismatches were present), which may reduce sensitivity. In any case, it seems likely that the efficacy of nUPDx is reduced for some pentastomes and that nUPDx may not detect others.

Another limitation of nUPDx is that the ∼200 base pair amplicon does not differentiate all parasites to the same taxonomic level which is related to the discriminatory potential of the target amplicon. For certain taxa (e.g., the human-infecting *Plasmodium* species) nUPDx offers excellent taxonomic resolution and should differentiate them to the species level (Flaherty et al., 2021). However, for other taxa, discrimination only to the level of genus or family is possible (e.g., certain filarial nematodes). Investigators should recognize this limitation prior to assigning an identity to a sequence detected via nUPDx. To better understand the discriminatory power of nUPDx, we curated our parasite sequence reference database (Supplementary File S2), so that sequences were named according to the most specific (i.e., lowest) taxonomic rank to which the sequence would obtain an identical match. As the full diversity of protozoan species infecting animals – particularly wildlife – worldwide remains opaque, the process of database curation is ongoing.

Evaluating the sensitivity of nUPDx based on the present dataset is challenging. When examining the mammalian specimens that were known to be positive for a parasitic infection, 75% tested positive via nUPDx. However, 5 of 13 (∼38%) of animals, primarily raccoons with *Babesia* spp. infections, that had previously tested negative via conventional PCR assays returned a positive nUPDx result. The use of archived samples from various hosts and pathogens that had been detected using different diagnostic methods to evaluate this assay is good in regards to evaluating the diversity of pathogens that can be detected, but is also problematic because samples were not obtained in a standardized manner and sample sizes are relatively small. It is likely that the sensitivity of this assay is higher for specific pathogen or host groups. Future efforts to improve nUPDx should focus on reducing the disparity between the number positive detections observed using other molecular tests (i.e., Table 1, Table 2) and the number of positives confirmed by nUPDx. However, the ability of nUPDx to detect infections that were previously overlooked is intriguing and highlights its potential diagnostic utility. Ultimately, nUPDx can detect these unanticipated infections because an a priori knowledge of a suspected pathogen is not required for nUPDx, in contrast to targeted PCR assays for example, which are only run if a particular etiological agent is suspected based on patient history.

We anticipate that with continued development, nUPDx will be useful in clinical diagnostic settings and/or as a screening tool for estimating the prevalence and identity of parasitosis in domestic animals and wildlife, with further application in wildlife surveillance for emerging parasitic diseases. The wide range of animal parasites detected here suggests that nUPDx could also prove useful for detecting and taxonomically classifying infections in humans caused by obscure zoonotic pathogens. In particular, this assay was useful for detection and classification of *Babesia* species (including coinfections) in multiple wildlife hosts. This is particularly relevant as new zoonotic *Babesia* species are being increasingly identified (Yabsley and Shock, 2013; Scott et al., 2021; Doderer-Lang et al., 2022) and certain hosts, like dogs and many wildlife species, harbor a diversity of *Babesia* species and coinfections can be common (Yabsley et al., 2006; Solano-Gallego et al., 2016; Fanelli, 2021). We confirm that nUPDx does not detect trichomonads and certain amoebae that are common pathogens for many animals, especially birds and reptiles, respectively. The inability to detect intestinal amoeba is a potential limitation of the assay particularly if testing fecal specimens, although in this study we only examined blood and solid tissues specimens. However, despite its limitations, nUPDx shows promise and with continued development will likely prove useful in both veterinary diagnostics and wildlife surveillance, especially because species-specific PCRs can miss unknown or unexpected pathogens.

## Disclaimer

The findings and conclusions in this report are those of the author(s) and do not necessarily represent the official position of the Centers for Disease Control and Prevention/the Agency for Toxic Substances and Disease Registry.

## Ethics

Ethics approval for the use of anonymized, de-identified, non-reidentifiable blood samples as non-engaged research was granted by Centers for Disease Control and Prevention Division of Parasitic Diseases and Malaria Human Subjects Review, approval number 2016–314. All animal DNA extracts utilized in this study were originally collected with approval obtained from the University of Georgia Institutional Animal Care and Use Committee (IACUC), animal use protocol (AUP) number A2018 02-010-Y2-A2.

## Author contributions

**ML:** all laboratory work, PCR, library preparation, sequencing, writing original drafts, tables, data analysis. **MK:** database curation, bioinformatic analysis, R code, figures. **JLNB:** bioinformatic analysis, R code, writing of original drafts, reviewing drafts, figures, tables, statistical analysis. **YQ:** project management, review of drafts. **MJY:** specimen collection, parasite identifications, review of drafts. **KBG:** specimen collection, parasite identifications, review of drafts. **RSB:** study conception, project management, review of drafts.

## Declaration of competing interest

The authors of this manuscript have no conflicts of interest to disclose. All funding sources are listed clearly in the acknowledgements section of this article.
